# Supplementary material for: Skeletal Muscle Nuclei in Mice are not Post-mitotic
Source: Function (Oxf). 2022 Nov 22;4(1):zqac059. doi: 10.1093/function/zqac059 (PMC9772608; doi:10.1093/function/zqac059)
Supplement: zqac059_Supplemental_File [file zqac059_supplemental_file.doc]

**Title: Skeletal muscle nuclei in mice are not post-mitotic**

Agnieszka K Borowik^1^, Arik Davidyan^1^, Frederick F Peelor III^1^, Evelina Voloviceva^1^, Stephen Doidge^1^, Matthew P Bubak^1^ Christopher B Mobley^2^, John J McCarthy^3,4^, Esther E Dupont-Versteegden^3,4,5^, Benjamin F Miller^1,6,7^

1. Aging and Metabolism Research Program, Oklahoma Medical Research Foundation, Oklahoma City, OK, USA

2. School of Kinesiology, Auburn University, Auburn, AL, USA

3. Center for Muscle Biology, University of Kentucky, Lexington, KY, USA

4. Department of Physiology, College of Medicine, University of Kentucky, Lexington, KY, USA

5. Department of Physical Therapy, College of Health Sciences, University of Kentucky, Lexington, KY, USA

6. Multiplexing Protein Analysis Core, Oklahoma Nathan Shock Center on Aging, Oklahoma City, OK, USA

7. Oklahoma City VA Medical Center, Oklahoma City, OK, USA

Corresponding Author:

Benjamin F Miller,

Aging and Metabolism Research Program

Oklahoma Medical Research Foundation

825 NE 13th Street

Oklahoma City, Oklahoma 73104

**Mouse Models**: The mouse models used for breeding are provided in **Supplementary Table 1**. The satellite cell specific conditional ablation mouse (Pax7-DTA) was generated as previously described^1^ by crossing Pax7CreER/CreER and Rosa26DTA/DTA strains. To generate the adult skeletal muscle myonuclear specific GFP mouse HSArtTA;Tet-O-H2B-GFP (HSA-GFP), we crossed the muscle‐specific Tet‐On (HSA‐rtTA) mouse^2^ with the tetracycline response element histone 2b green fluorescent protein (TRE‐H2B‐GFP) mouse^3^. To generate the Pax7-DTA;HSA-GFP mouse we first crossed, through two rounds of breeding, the Pax7CreER/CreER to HSA-rtTA and the Rosa26DTA/DTA to the TetO-H2B-GFP to generate Pax7CreER/CreER; HSA-rtTA and Rosa26DTA/DTA;TetO-H2B-GFP, respectively. These two strains were then crossed to generate the Pax7-DTA;HSA-GFP mouse.

**Supplementary Table 1**: List of used animal models in the study.

**Genotyping:** DNA was extracted from tails, and 2 μl (5-20 ng) was used in the subsequent PCR reaction. Each 25-μl PCR reaction contained Green GoTaq Master mix (Promega), 0.5 μM primers and nuclease free water. Primer sequences and used PCR conditions are provided in **Supplementary Table 2**. The Pax7-DTA;HSA-GFP: We used a two-factor validation approach, which includes SYBR based PCR followed by agarose gel PCR. Agarose gel PCR was used to confirm genotype of HSA-rtTA and H2B-GFP as described in HSA-GFP mice.

**Supplementary Table 2**: List of primer sequences and PCR conditions used for mice genotyping.

**Supplementary Table 3:** Detailed information about animals used in the study.

**Supplementary Table 4**: Antibodies used for immunohistochemistry analysis and validation of HSA-GFP skeletal muscles.

**
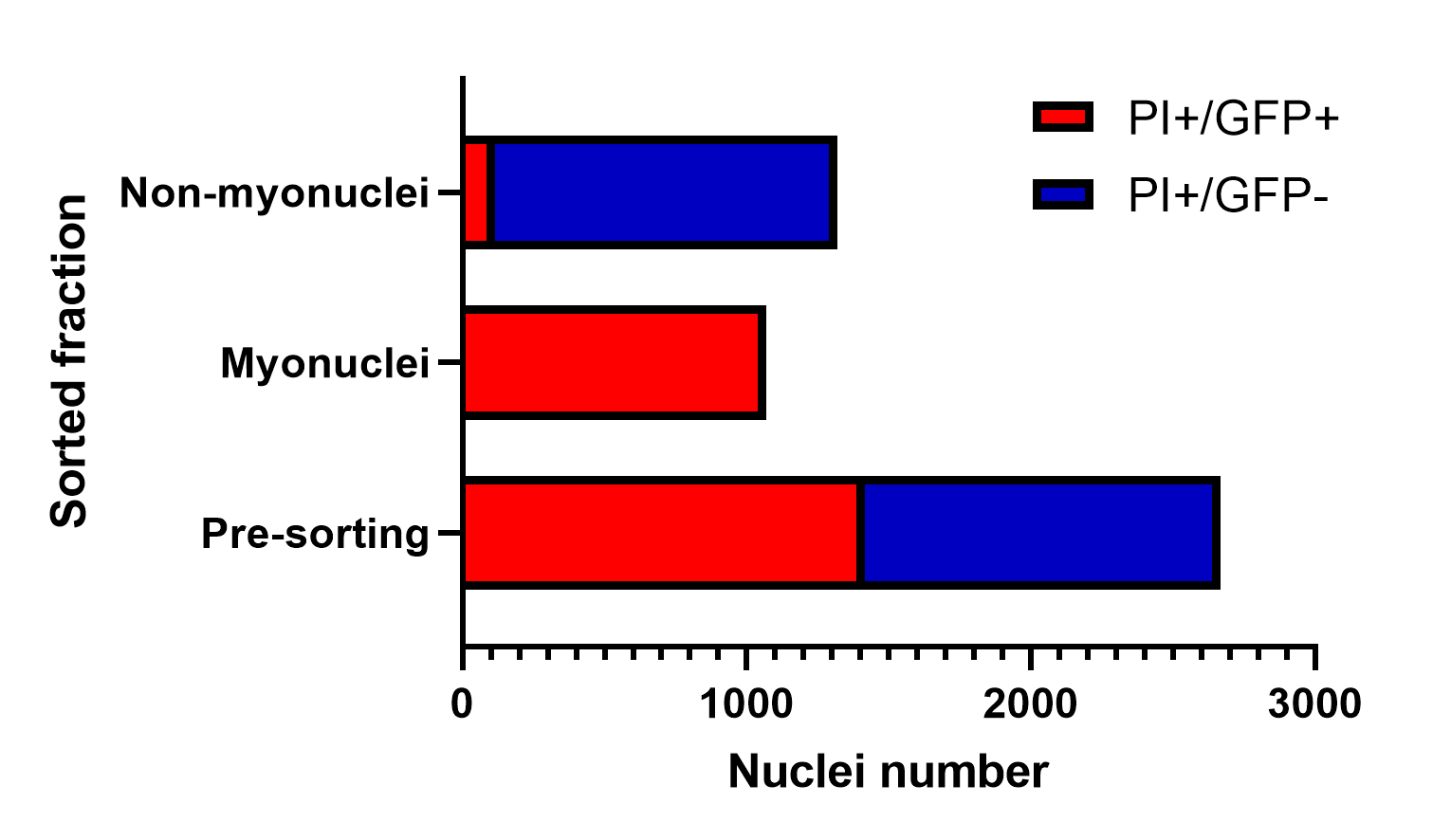
**

**Supplementary Figure 1: Representative quantification of the nuclei sorting efficiency.** The aliquots of crude nuclear fraction before FACS sorting, as well as sorted myonuclear and non-myonuclear fractions were analyzed for their composition. The pre-sorted fraction contained 1416 PI^+^/GFP^+^ nuclei (myonuclei) and 1252 PI^+^/GFP^-^ nuclei coming form the other cell types. After sorting, myonuclear fraction had only PI^+^/GFP^+^ nuclei (1071 counts), while non-myonuclear fraction consisted of 114 PI^+^/GFP^+^ nuclei and 1205 PI^+^/GFP^-^ nuclei.

**Supplementary Table 5: Calculations for DNA fraction synthesis rates in nuclei from HSA-H2B mice kept in normal cage conditions.**Data were registered using unlabeled animal corrections, and bone marrow was used as a fully turned over pool.

**Supplementary Table 6: Calculations for DNA fraction synthesis rates in nuclei from HSA-H2B mice kept in normal cage conditions.**Data were registered using unlabeled animal corrections, and mass isotopomer distribution analysis (MIDA) adjustment was used.

**Supplementary Table 7: Calculations for DNA fraction synthesis rates in nuclei from HSA-H2B mice after functional overload.**Data were registered using unlabeled animal corrections, and bone marrow was used as a fully turned over pool.

**Supplementary Table 8: Calculations for DNA fraction synthesis rates in nuclei from HSA-H2B mice after functional overload.**Data were registered using unlabeled animal corrections, and mass isotopomer distribution analysis (MIDA) adjustment was used.

**Supplementary Table 9: Calculations for DNA fraction synthesis rates in nuclei from HSA-H2B mice after satellite cells ablation.**Data were registered using unlabeled animal corrections, and bone marrow was used as a fully turned over pool.

**Supplementary Table 10: Calculations for DNA fraction synthesis rates in nuclei from HSA-H2B mice after satellite cells ablation.**

Data were registered using unlabeled animal corrections, and mass isotopomer distribution analysis (MIDA) adjustment was used.

**Supplementary Table 11: Calculations for DNA fraction synthesis rates in nuclei from HSA-H2B mice kept in normal cage conditions.**Data were registered using standard curve corrections, and bone marrow was used as a fully turned over pool.

**Supplementary Table 12: Calculations for DNA fraction synthesis rates in nuclei from HSA-H2B mice kept in normal cage conditions.**Data were registered using standard curve corrections, and mass isotopomer distribution analysis (MIDA) adjustment was used.

**Supplementary Table 13: Calculations for DNA fraction synthesis rates in nuclei from HSA-H2B mice after functional overload.**Data were registered using standard curve corrections, and bone marrow was used as a fully turned over pool.

**Supplementary Table 14: Calculations for DNA fraction synthesis rates in nuclei from HSA-H2B mice after functional overload.**Data were registered using standard curve corrections, and mass isotopomer distribution analysis (MIDA) adjustment was used.

**Supplementary Table 15: Calculations for DNA fraction synthesis rates in nuclei from HSA-H2B mice after satellite cells ablation.**Data were registered using standard curve corrections, and bone marrow was used as a fully turned over pool.

**Supplementary Table 16: Calculations for DNA fraction synthesis rates in nuclei from HSA-H2B mice after satellite cells ablation.**Data were registered using standard curve corrections, and mass isotopomer distribution analysis (MIDA) adjustment was used.


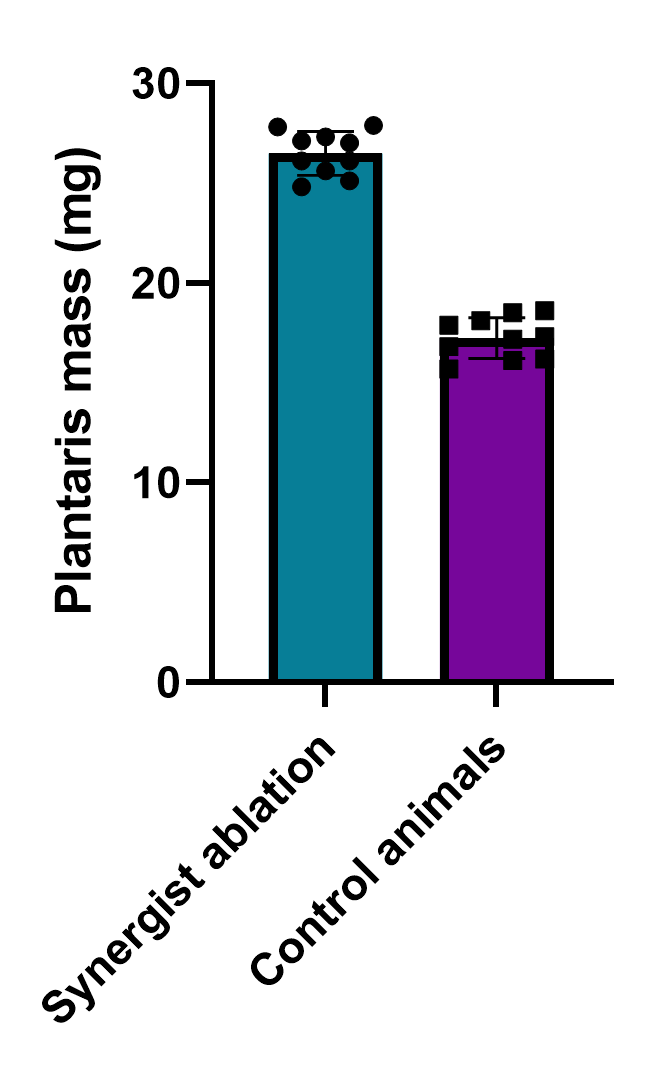


**Supplementary Figure 2: Plantaris muscle mass increased after synergist ablation when compared to control animals.** 8 weeks after synergist ablation, overloaded plantaris muscles mass from HSA-H2B mice was 26.48 ± 1.10 mg. The mass of plantaris muscles collected from age-matching control animals was 17.24 ± 1.03 mg. Values are presented as mean ± standard deviation (SD).

**
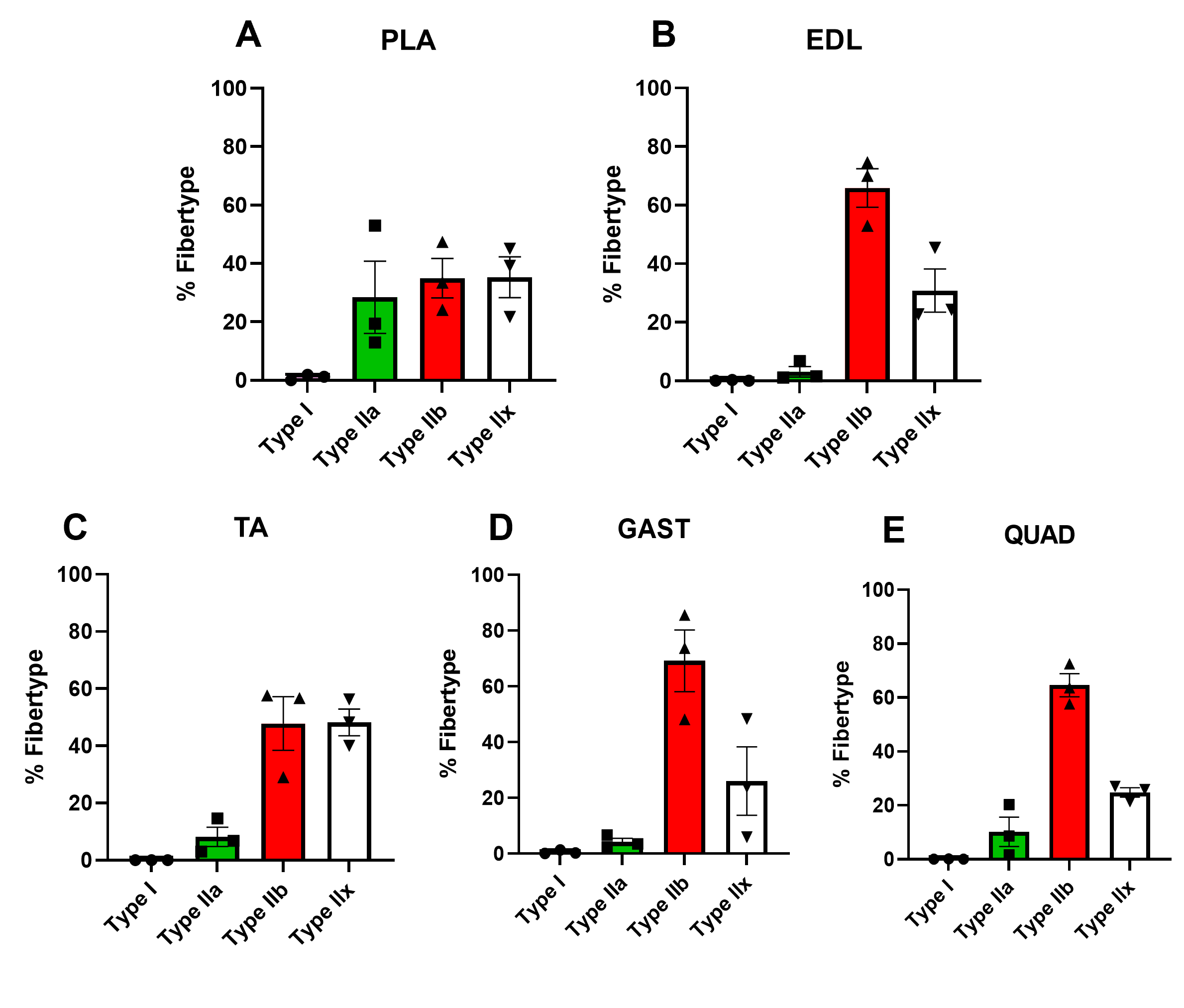
**

**Supplementary Figure 3: Immunohistochemical identification of HSA-H2B muscle fiber types.**  Proportion of fibers in plantaris PLA **(A)**, extensor digitorum longus EDL **(B)**, tibialis anterior TA **(C)**, gastrocnemius GAST **(E)**, quadriceps QUAD **(F)** skeletal muscles.


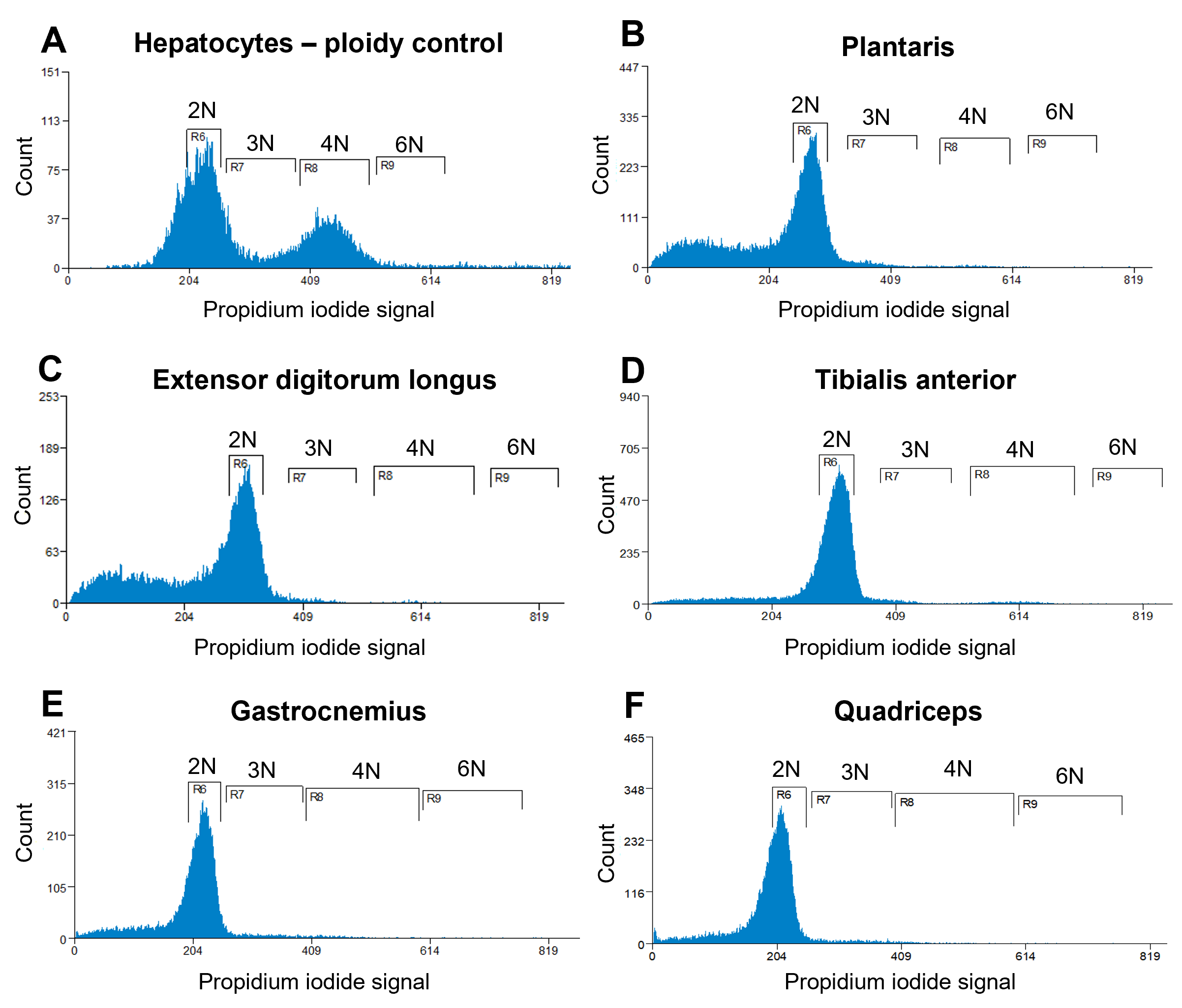


**Supplementary Figure 4: Representative analysis of ploidy levels in sorted myonuclei from muscles from animals kept in normal cage conditions.** Cell flow cytometry of hepatocytes **(A)**, myonuclei isolated from plantaris **(B)**, extensor digitorum longus **(C)**, tibialis anterior **(D)**, gastrocnemius **(E)**, quadriceps **(F)**. The peaks corresponding to diploid nuclei are labeled 2N, triploid nuclei as 3N, tetraploid nuclei as 4N and hexaploid nuclei as 6N.


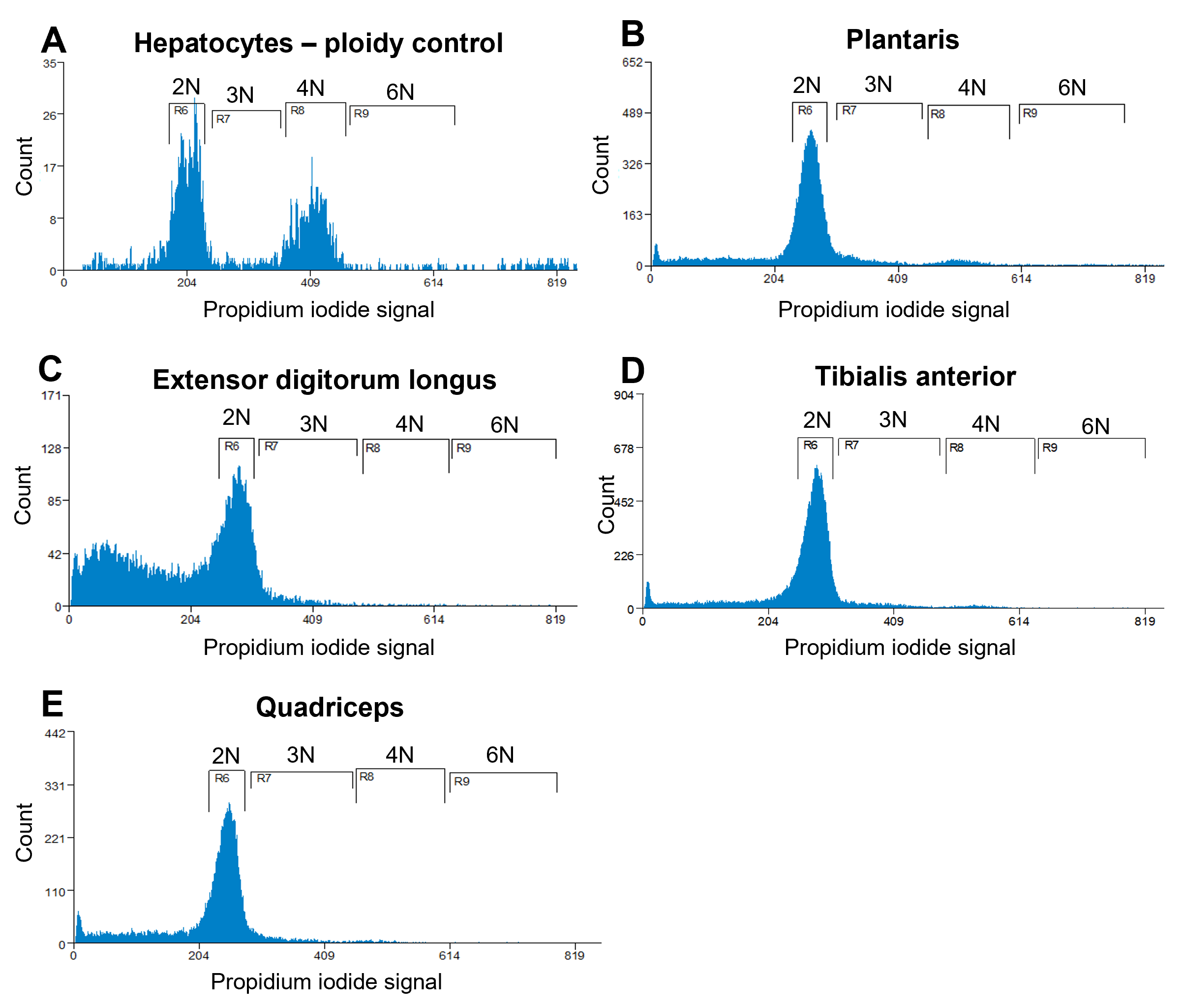


**Supplementary Figure 5: Representative analysis of ploidy levels in sorted myonuclei from muscles from animals with functional overload.** Cell flow cytometry of hepatocytes **(A)**, myonuclei isolated from plantaris **(B)**, extensor digitorum longus **(C)**, tibialis anterior **(D)**, quadriceps **(E)**. The peaks corresponding to diploid nuclei are labeled 2N, triploid nuclei as 3N, tetraploid nuclei as 4N and hexaploid nuclei as 6N.


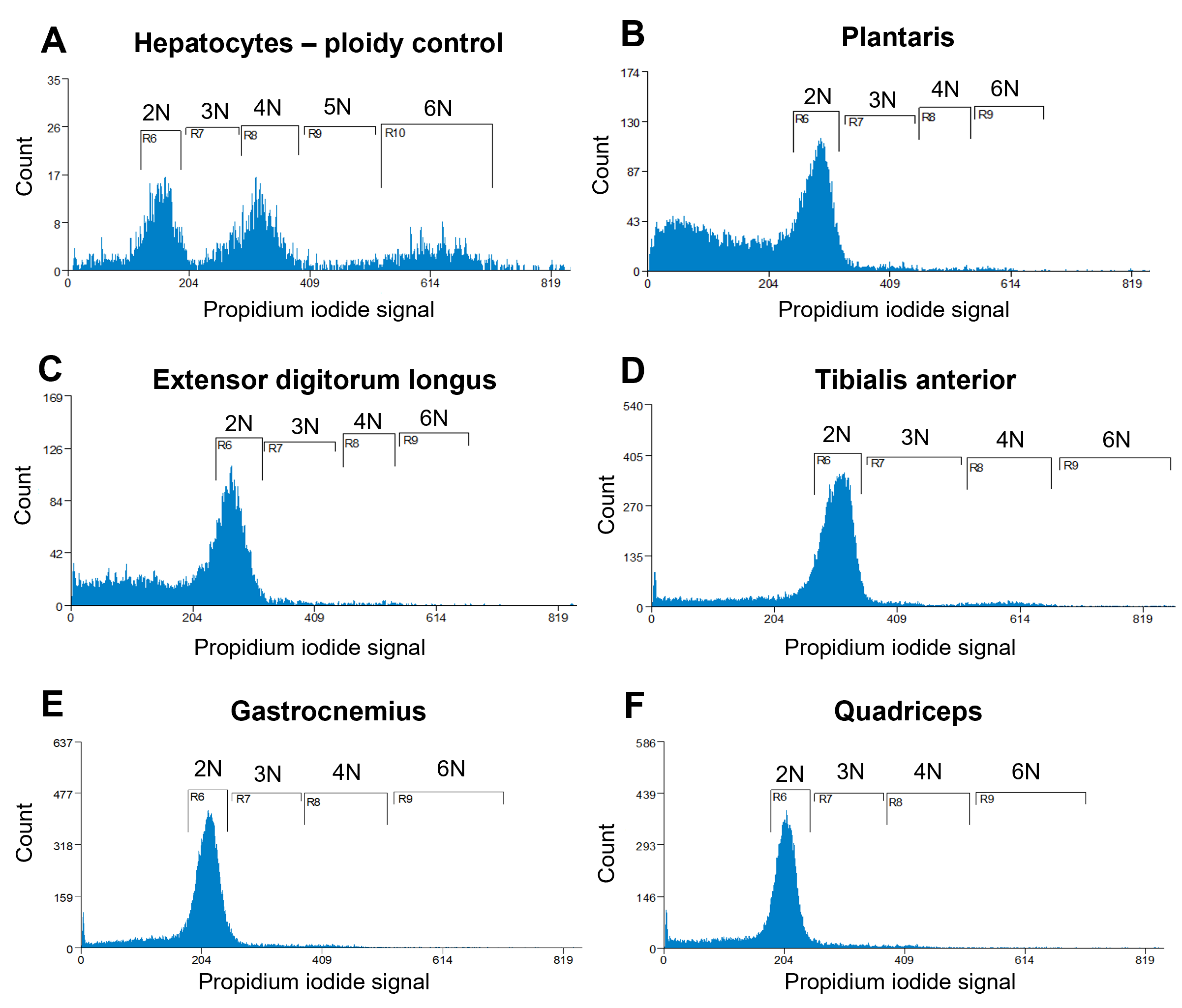


**Supplementary Figure 6: Representative analysis of ploidy levels in sorted myonuclei from muscles from animals after satellite cells ablation.** Cell flow cytometry of hepatocytes **(A)**, myonuclei isolated from plantaris **(B)**, extensor digitorum longus **(C)**, tibialis anterior **(D)**, gastrocnemius **(E)**, quadriceps **(F)**. The peaks corresponding to diploid nuclei are labeled 2N, triploid nuclei as 3N, tetraploid nuclei as 4N, pentaploid nuclei as 5N and hexaploid nuclei as 6N.

**References**

1. Mccarthy JJ, Mula J, Miyazaki M, et al. Effective fiber hypertrophy in satellite cell-depleted skeletal muscle. *Development*. 2011;138(17):3657-3666.

2. Iwata M, Englund DA, Wen Y, et al. A novel tetracycline-responsive transgenic mouse strain for skeletal muscle-specific gene expression. *Skelet Muscle*. 2018;8(1):1-8.

3. Tumbar T, Guasch G, Greco V, et al. Defining the epithelial stem cell niche in skin. *Science*. 2004;303(5656):359-363.
